# Analysis of a heterogeneous functionally graded material with a spherical void exposed to time-dependent ramp-type heating according to the TPL heat conduction model

**DOI:** 10.1038/s41598-025-11471-3

**Published:** 2025-07-24

**Authors:** Sami F. Megahid

**Affiliations:** 1 Department of Mathematics, Faculty of Science, Mansoura University, Mansoura, 35516 Egypt; 2 Department of Mathematics, Faculty of Science, New Mansoura University, New Mansoura City, 35712 Egypt

**Keywords:** Thermo-elastic functionally graded materials, Spherical hole, Non-homogeneity, TPL heat equation, Time-dependent slope-type heating, Materials science, Mathematics and computing

## Abstract

Functionally graded materials (FGMs) are sophisticated composites distinguished by a progressive alteration in composition and characteristics, facilitating customized performance for particular applications. Their distinctive architecture facilitates improved mechanical characteristics, thermal resilience, and operational efficacy in domains such as aerospace, biomedical, and automotive engineering. This study presents a novel three-phase-lag (TPL) thermal conductivity model and examines the thermoelastic behavior of a functionally graded medium featuring a spherical void. The paper examines the coupled thermo-mechanical behavior under time-dependent slope-type heating given to the sphere’s traction-free inner surface, addressing complex linkages often overlooked in previous research. This research meticulously investigates the influence of critical factors, relaxation times, and ramping time on the dynamic physical response. A rigorous Laplace transform methodology is used to resolve the governing equations, which yields extensive quantitative insights. The findings indicate that the dynamic behavior of functionally graded materials under complex mechanical and thermal loading situations can be more comprehensively understood. This study makes a significant contribution to the field by integrating the TPL model with functionally graded analysis. This represents a substantial progression in thermoelastic functionally graded analysis, with prospective applications in the design of advanced materials, optimization of thermal management systems, and smart material technologies.

## Introduction

Many scientific and engineering applications depend significantly on the principles of thermoelasticity and thermal conduction in elastic materials. An interdisciplinary approach enables the regulation of the mechanical and thermal properties of materials, leading to precise predictions and enhanced designs. This collaboration facilitates the design and enhancement of buildings and technology across several sectors^1–3^. Integrating thermal conduction and thermoelasticity is essential for developing new materials and analyzing thermal stresses in engineering components, since it provides a thorough understanding of material responses to thermal loading^4^.

A family of advanced composite materials termed functionally graded materials (FGMs) is characterized by a gradual variation in composition and properties throughout its volume. FGMs are particularly advantageous for use in extreme settings, such as biomedical engineering, automotive, and aerospace, due to this innovative approach that enhances mechanical and thermal properties. The notion of FGMs is inspired by natural materials with graded structures, such as bones and teeth, which have developed for maximum performance under particular loading circumstances^5^.

The continuous gradient in material composition employed in the design of FGMs facilitates tailored mechanical and thermal properties. A variety of production procedures can be employed to achieve this gradation, enabling the creation of materials that are resistant to mechanical stresses and significant temperature variations. In comparison to traditional materials, FGMs provide several advantages owing to their unique structure. For instance, they can enhance overall performance in challenging environments, mitigate thermal stressors, and augment wear resistance. Consequently, they are ideal for applications such as biomedical implants, thermal barrier coatings, and turbine blades^6^.

FGMs are produced by many methods, including chemical vapor deposition, additive manufacturing, and powder metallurgy. Powder metallurgy provides precise control over the composition and properties of a material by amalgamating powders of diverse substances and sintering them to form a solid. Additive manufacturing, including 3D printing, enables the production of FGMs with intricate designs, facilitating the fabrication of complicated geometries with tailored gradients. Chemical vapor deposition is commonly employed in coatings and microelectronics to generate thin material layers with controlled gradients^7^.

FGMs serve several functions. They are employed in aviation components, including turbine blades and thermal barrier coatings, to endure elevated temperatures and mechanical stresses. FGMs are utilized in biomedical engineering for implants and prostheses because of their varied properties, which enhance biocompatibility and mimic the natural architecture of bone. FGMs are utilized in energy systems such as batteries and fuel cells, enhancing efficiency and temperature regulation^5–7^.

The primary objectives of current research in the domain of FGMs are to improve fabrication techniques, understand thermomechanical behavior under diverse stress conditions, and explore innovative applications. The progression of FGM application in high-performance engineering contexts is contingent upon this research. Researchers aim to enhance the resilience and reliability of FGMs under practical conditions by optimizing the gradient of material properties^8–11^.

The thermo-elastic behavior of transversely isotropic functionally graded materials with hollow spheres has not been thoroughly investigated, particularly concerning their reaction to sudden mechanical loads or arbitrary thermal loads. This gap underscores the need for further research into the response of functionally graded structures to dynamic conditions, enhancing our understanding and application across several engineering fields.

Hooke’s law of elasticity and Fourier’s heat conduction equation serve as the foundation for conventional thermoelasticity (CTE), sometimes referred to as the linear theory of thermoelasticity^12^. According to CTE, heat transfer occurs instantaneously, and mechanical and thermal impacts spread equally. The CTE hypothesis predicts infinitely high wave velocities, which will cause a quick thermal response, as multiple studies have shown. This prediction does, however, occasionally diverge from real, observable events and empirical realities.

In order to overcome the restriction of infinite heat propagation speed and develop a framework for modeling heat transfer with constrained propagation velocities, Lord and Shulman significantly improved Fourier’s law by adding thermal relaxation time^13^. In order to provide a more nuanced depiction of heat transport dynamics and to capture intricate thermal processes, Green and Lindsay suggested an improved model that incorporates two thermal relaxation durations^14^. By classifying material responses into three different models according to heat conduction properties, Green and Naghdi expanded the theoretical framework^15–17^: Type I, which follows classical heat conduction laws; Type II, which describes heat propagation at finite speeds without dissipation; and Type III, which incorporates thermal damping effects while paradoxically predicting infinite heat propagation speed.

The topic of thermoelasticity has significantly advanced due to recent advancements by Tzou^18,19^ and Choudhuri^20^who have included microstructural effects into conventional heat transfer models. By examining the impact of microscale phenomena on material behavior, Tzou’s dual-phase-lag model (DPL) shows how these variables change mechanical and thermal properties. By combining thermal processes and mechanical reactions, his research increases the precision of thermal stress prediction in a range of situations. In order to enhance Fourier’s law, Choudhuri developed a three-phase-lag model (TPL), which incorporates temporal lags in thermal displacement, temperature fluctuations, and heat transfer. Heat diffusion models’ forecast accuracy is increased by this novel approach, which makes them especially appropriate for complex systems and circumstances characterized by abrupt or severe thermal fluctuations.

Advanced thermoelasticity frameworks known as the three-phase-lag (TPL) and dual-phase-lag (DPL) models take into account time delays in the movement of mechanical waves and heat that are not taken into account by more traditional models such as Fourier’s law. The main distinction between the two is how many phase delays they take into account for different physical properties associated with deformation and heat transmission. The phase lag components and applications of the DPL and TPL models are essentially different. Heat flux and temperature gradient are the two phase lags included in the DPL model, but a third lag for the thermal displacement gradient is added in the TPL model. The TPL model’s applicability in complex situations is enhanced by this extra element, especially in functionally graded materials and nanoscale systems where thermal-mechanical interaction is crucial. When thermal deformation effects are negligible, the DPL model continues to work well for typical heat transfer applications due to its simplicity.

By incorporating limited heat propagation velocities within materials, the three-phase-lag (TPL) model outperforms the traditional Fourier law. Accurate modeling of heat transfer in complicated circumstances is made easier by integrating time delays in temperature gradient, thermal displacement gradient, and heat flux. Because of this, it is very useful for studying materials with heterogeneous characteristics and nonlocal impacts, especially in cutting-edge applications like microelectronics and aerospace. The TPL model provides a more accurate representation of heat transfer, particularly in materials undergoing rapid thermal fluctuations, by incorporating temporal delays in heat flux, temperature gradient, and thermal displacement gradient.

Generalized thermoelasticity models effectively tackle the issue of thermal waves by accurately predicting that thermal signals propagate at finite velocities. These models are highly proficient in simulating heat conduction in variable temperature fields. The generalized thermoelasticity models, as demonstrated in^21–37^proficiently address the heat wave problem by providing a more accurate depiction of thermal conductivity.

This study aims to introduce a more comprehensive and refined framework for thermoelasticity that more precisely represents the correlation between temperature and elasticity, in contrast to past models. This new model integrates the three-phase-lag (TPL) equation with the Lord–Shulman, dual-phase-lag (DPL), and Green–Naghdi type III (GN-III) thermoelastic theories. Relaxation times are integrated into the improved model to more accurately reflect the complex responses of functionally graded materials to thermal expansion and heating.

The study’s capacity to accurately represent the behavior of more intricate functionally graded materials distinguishes the model from earlier generalized thermoelasticity models, constituting one of its primary contributions. The proposed paradigm offers enhanced predictions and insights into the thermoelastic response of functionally graded materials, elucidating the intricate relationship between temperature and elasticity.

A thermoelastic unconfined heterogeneous transversely isotropic functionally graded media with a spherical cavity subjected to time-dependent slope-type heating is modeled directly using the TPL thermal model. Inverse transformations are computed using the Laplace transform and numerical algorithm methods to analyze this system. The system’s behavior is analyzed in several ways, encompassing both graphical and analytical techniques. The physical properties of titanium alloy are utilized in numerical calculations. The study incorporates significant variables, such as the effects of thermal relaxation durations and ramping time. The findings of this work hold significant potential for facilitating the development of analytical tools and optimization methods tailored for heterogeneous functionally graded systems.

This discovery could have substantial implications for engineering, materials science, and structural analysis, among other fields. Facilitating the design and evaluation of diverse systems and structures subjected to thermal and mechanical loads can ultimately enhance efficiency, safety, and durability.

## Fundamental equations

The governing equations for thermoelasticity theory in functionally graded materials subjected to a thermal field can be formulated utilizing the three-phase-lag thermal conductivity model for a transversely isotropic medium^38–41^:

The updated TPL heat formula

1 $$\:\left(1+{\tau\:}_{q}\:\frac{\partial\:}{\partial\:t}\right)\overrightarrow{q}=-K\left(1+{\tau\:}_{\theta\:}\:\frac{\partial\:}{\partial\:t}\right)\overrightarrow{\nabla\:}\theta\:-{K}^{\text{*}}\left(1+{\tau\:}_{\psi\:}\:\frac{\partial\:}{\partial\:t}\right)\overrightarrow{\nabla\:}\psi\:$$

The energy balance formula

2 $$\:\frac{\partial\:}{\partial\:t}[\rho\:{C}_{e}\theta\:+{T}_{0}{\beta\:}_{ij}{S}_{ij}]=-\overrightarrow{\nabla\:}\bullet\:\overrightarrow{q}+Q$$

The relationships between stress and strain

3 $$\:{\tau\:}_{ij}={C}_{ijkl}{S}_{kl}-{\beta\:}_{ij}\theta\:.$$

The connection between displacement and strain

4 $$\:{S}_{ij}=\frac{1}{2}\left({u}_{j,i}+{u}_{i,j}\right).$$

The equation of motion

5 $$\:{\tau\:}_{ij,j}=\rho\:{\ddot{u}}_{i}$$

By using Eq. (3) to replace $\:{\tau\:}_{ij}$ in Eq. (5), the following outcome can be obtained:

6 $$\:{\left({C}_{ijkl}{S}_{kl}\right)}_{,j}-{\left({\beta\:}_{ij}\theta\:\right)}_{,j}=\rho\:{\ddot{u}}_{i}$$

$\:\overrightarrow{q}$ represents heat flow, $\:\theta\:=T-{T}_{0}$ for temperature change, $\:T$ for absolute temperature, and $\:{T}_{0}$ for ambient temperature in Eq. (1) through (6). $\:{u}_{i}$ stands for displacements, $\:{C}_{e}$ for specific heat, $\:Q$ for heat supply, $\:\rho\:$ for density, $\:{\tau\:}_{ij}$ for stress tensor, $\:{S}_{ij}$ for strain tensor, and $\:K$ for thermal conductivity. $\:{\beta\:}_{ij}={C}_{ijkl}{\alpha\:}_{kl}$ is a formula where $\:{\alpha\:}_{kl}$ is the thermal expansion tensor and $\:{C}_{ijkl}$ is an isothermal elastic tensor. The thermal-displacement scalar function $\:\psi\:$ satisfies $\:\dot{\psi\:}=\theta\:$, and $\:{K}^{\text{*}}$ is a material constant that is a component of the Green and Naghdi theories and is referred to as the thermal conductivity rate. Furthermore, the relaxation time in temperature gradient is represented by $\:{\tau\:}_{\theta\:}$, the relaxation time in heat flux by $\:{\tau\:}_{q}$, and the relaxation time in thermal displacement gradient by $\:{\tau\:}_{\psi\:}$.

If $\:\overrightarrow{q}$ is taken out of Eqs. (1) and (2) and $\:\dot{\psi\:}=\theta\:$ is put in, the modified heat transfer three-phase-lag equation can be differentiated with respect to time and represented as follows:

7 $$\:\begin{array}{c}\left(1+{\tau\:}_{q}\:\frac{\partial\:}{\partial\:t}\right)\left[\frac{\partial\:}{\partial\:t}\left(\rho\:{C}_{e}\frac{\partial\:\theta\:}{\partial\:t}\right)+{T}_{0}\frac{{\partial\:}^{2}}{\partial\:{t}^{2}}\left({\beta\:}_{ij}{S}_{ij}\right)-\frac{\partial\:Q}{\partial\:t}\right]=\\\:\left(1+{\tau\:}_{\theta\:}\:\frac{\partial\:}{\partial\:t}\right)\nabla\:.\left(K\nabla\:\dot{\theta\:}\right)+\left(1+{\tau\:}_{\psi\:}\:\frac{\partial\:}{\partial\:t}\right)\nabla\:.\left({K}^{\text{*}}\nabla\:\theta\:\right).\end{array}$$

With the equation of motion (6) and the appropriate boundary and beginning conditions, the modified thermal transfer Eq. (7) is frequently used to solve certain thermoelasticity problems, such as examining heat dispersion in a rod, plate, cylinder, or sphere. This model’s features improve our comprehension of thermal dynamics while also enabling more precise predictions in a variety of applications. These applications include thermomechanical systems, high-speed heat transmission, and materials research in engineering settings.

### Application

**Fig. 1:**
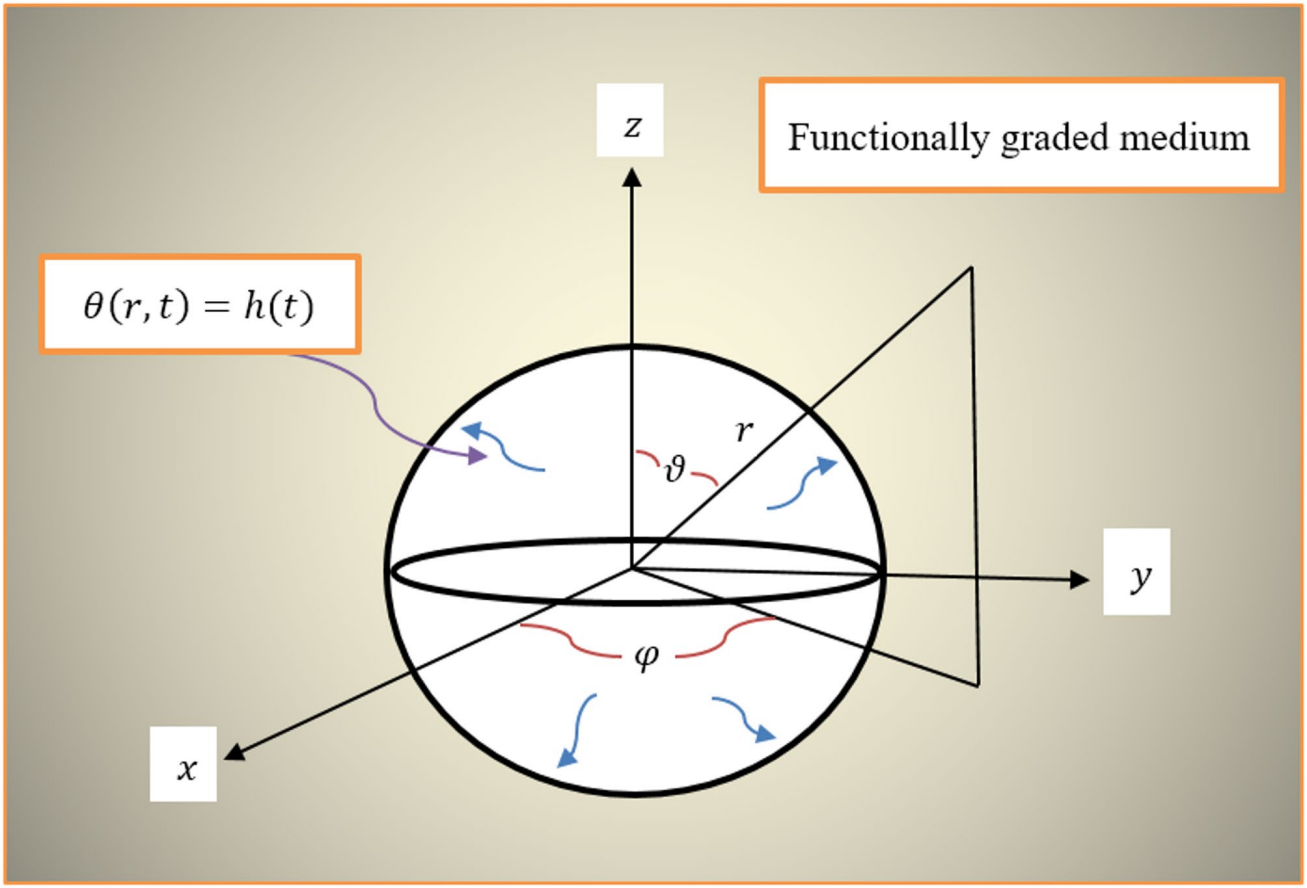
Schematic diagram of thermoelastic functionally graded medium with spherical void.

As an application of the novel theory, this section will discuss the problem of an endlessly flexible, heterogeneous, transversely isotropic, thermally functionally graded material with a spherical void of radius $\:a$ (see Fig. 1). Verifying the accuracy of the suggested model is the aim of this investigation. We assume spherical symmetry of the interactions and place the origin of the cavity in the center using spherical polar coordinates $\:\left(r,\vartheta\:,\phi\:\right)$. Therefore, the radial distance $\:r$ and time $\:t$ are the only factors that affect any interaction. For this spherically symmetric problem, the focus will be on the radial displacement component, represented as $\:{u}_{r}=u\left(r,t\right)$.

Isotropic thermoelastic materials display consistent properties in all directions, indicating that their mechanical and thermal responses are homogeneous irrespective of orientation. This simplicity is advantageous for numerous applications involving uniform materials, facilitating direct analysis and design. Transversely isotropic thermoelastic materials exhibit isotropic qualities in one plane while demonstrating variation along a perpendicular axis. This indicates that their responses to stress and temperature fluctuations vary depending on the orientation. The presumption of transverse isotropy is essential for precisely simulating the behavior of composite materials that exhibit varying mechanical characteristics in different directions.

Limiting radial variations in functionally graded materials (FGMs) is crucial for streamlining the investigation and modeling of their behavior under diverse loading circumstances. This methodology enables engineers and researchers to concentrate on radial symmetry, thereby simplifying intricate equations and aiding in the derivation of analytical solutions. Radial variations are extremely pertinent in numerous practical applications, particularly those involving cylindrical or spherical geometries, such as turbine blades or pressure vessels. By customizing material qualities radially, designers can improve performance, optimize heat resistance, and maintain structural integrity without the complications of angular changes.

The formulation addresses boundary conditions for a spherical cavity experiencing time-dependent slope-type heating in the absence of external traction. This boundary condition replicates realistic thermal loading events, including abrupt temperature fluctuations resulting from environmental influences or operational circumstances. It facilitates the analysis of transitory thermal phenomena that are essential in aerospace and automotive engineering. The lack of external traction streamlines the model, concentrating exclusively on thermal effects and facilitating a more lucid examination of the thermal response. Therefore, the following formula can be used to define the boundary conditions at the cavity’s interior surface:

8 $$\:\begin{array}{c}{\tau\:}_{rr}\left(r,t\right)=0\:\:\:\:\:\:\:\:\:\:\:\:\:\text{a}\text{t}\:\:\:\:\:\:\:\:r=a,\:\:\:\\\:\theta\:\left(r,\:t\right)=h\left(t\right)={\theta\:}_{0}\left\{\begin{array}{c}\frac{t}{{t}_{r}}\:\:\:\:\:\:\:\:\:if\:\:\:\:\:\:\:0\le\:t\le\:{t}_{r}\\\:1\:\:\:\:\:\:\:\:\:if\:\:\:\:\:\:\:\:t>{t}_{r}\:\:\:\:\:\:\:\:\end{array}\right.\:\:\:\:\:\:\text{a}\text{t}\:\:\:\:r=a.\end{array}$$

In this context, $\:{t}_{r}$ denotes the ramping time parameter, while $\:{\theta\:}_{0}$, a positive constant, indicates the intensity of thermal loading.

For a spherically transversely isotropic functionally graded material, the constitutive relations are as follows^40,41^:

9 $$\:{\tau\:}_{rr}={C}_{11}{S}_{rr}+{C}_{12}\left({S}_{\vartheta\:\vartheta\:}+{S}_{\phi\:\phi\:}\right)-{\beta\:}_{11}\theta\:,$$

10 $$\:{\tau\:}_{\phi\:\phi\:}={\tau\:}_{\vartheta\:\vartheta\:}={C}_{12}{S}_{rr}+{C}_{22}{S}_{\vartheta\:\vartheta\:}+{C}_{23}{S}_{\phi\:\phi\:}-{\beta\:}_{22}\theta\:,$$

with

11 $$\:{\beta\:}_{11}={\alpha\:}_{1}{C}_{11}+2{\alpha\:}_{2}{C}_{12},\:\:{\beta\:}_{22}={\alpha\:}_{1}{C}_{12}+{\alpha\:}_{2}{C}_{22}+{\alpha\:}_{2}{C}_{23},$$

where $\:{\alpha\:}_{1}$ and $\:{\alpha\:}_{2}$ are the parameters that represent the coefficients of thermal expansion. To simplify, $\:{C}_{1111}$, $\:{C}_{2222}$, $\:{C}_{1122}$, $\:{C}_{2233}$, $\:{\alpha\:}_{11}$, and $\:{\alpha\:}_{22}$ are denoted by $\:{C}_{11}$, $\:{C}_{22}$, $\:{C}_{12}$, $\:{C}_{23}$, $\:{\alpha\:}_{1}$, and $\:{\alpha\:}_{2}$.

The following expression can be used to represent the strains for the spherically symmetric problem^40,41^:

12 $$\:{S}_{rr}=\frac{\partial\:u}{\partial\:r}\:\:,\:{S}_{\vartheta\:\vartheta\:}={S}_{\phi\:\phi\:}=\frac{u}{r},\:\:{S}_{r\vartheta\:}={S}_{r\phi\:}={S}_{\vartheta\:\phi\:}=0.$$

By substituting Eq. (12) into the governing Eqs. (9) and (10), the following formulas are obtained:

13 $$\:{\tau\:}_{rr}={C}_{11}\frac{\partial\:u}{\partial\:r}+{C}_{12}\frac{2u}{r}-{\beta\:}_{11}\theta\:,$$

14 $$\:{\tau\:}_{\phi\:\phi\:}={C}_{12}\frac{\partial\:u}{\partial\:r}+\left({C}_{22}+{C}_{23}\right)\frac{u}{r}-{\beta\:}_{22}\theta\:.$$

It is possible to write the equation of motion as

15 $$\:\frac{\partial\:{\tau\:}_{rr}}{\partial\:r}+\frac{2}{r}\left({\tau\:}_{rr}-{\tau\:}_{\phi\:\phi\:}\right)=\rho\:\frac{{\partial\:}^{2}u}{\partial\:{t}^{2}}.$$

In the absence of heat supply $\:(Q=0)$, one way to express the TPL heat equation is as

16 $$\:\begin{array}{c}\left(1+{\tau\:}_{q}\:\frac{\partial\:}{\partial\:t}\right)\left[\rho\:{C}_{e}\frac{{\partial\:}^{2}\theta\:}{\partial\:{t}^{2}}+{T}_{0}\frac{{\partial\:}^{2}}{\partial\:{t}^{2}}\left({\beta\:}_{11}\frac{\partial\:u}{\partial\:r}+{\beta\:}_{22}\frac{2u}{r}\right)\right]=\\\:\left(1+{\tau\:}_{\theta\:}\:\frac{\partial\:}{\partial\:t}\right)\nabla\:.\left(K\nabla\:\dot{\theta\:}\right)+\left(1+{\tau\:}_{\psi\:}\:\frac{\partial\:}{\partial\:t}\right)\nabla\:.\left({K}^{\text{*}}\nabla\:\theta\:\right).\end{array}$$

### Mathematical simulation of FGM in spherical frameworks

Functionally graded materials (FGMs) are transforming materials engineering by facilitating deliberate alterations to a material’s composition. This tailored technique enables engineers to fulfill specific practical requirements, enhancing durability, efficiency, and functionality in challenging conditions. FGMs exhibit significant alterations in characteristics, such as density, isothermal elastic tensor, and thermal conductivity, in contrast to conventional composites. This gradient modifies its physical and thermal properties to meet certain operational requirements.

The selection of material gradation is crucial and can be represented by either power-law or exponential variation. The power-law model is preferred due to its simplicity and capacity to depict progressive changes in material properties, rendering it appropriate for numerous engineering applications. In contrast, the exponential model provides enhanced flexibility, especially for materials that demonstrate swift property alterations, thereby representing more intricate behaviors in specific applications.

The characteristics of functionally graded materials (FGMs) can be quantitatively articulated as functions of the position $\:r$ in engineering analysis, particularly concerning spherical components like spherical voids subjected to thermal limitations. The subsequent formulas present an expression for this relationship^28^

17 $$\:{C}_{ij}={h}_{ij}{\left(\frac{r}{a}\right)}^{l},\:\rho\:={\rho\:}_{h}{\left(\frac{r}{a}\right)}^{l},\:\:K={K}_{h}{\left(\frac{r}{a}\right)}^{l},\:\:{K}^{*}={K}_{h}^{*}{\left(\frac{r}{a}\right)}^{l},$$

where $\:l$ represents the heterogeneity factor. Functionally graded materials (FGMs) frequently exploit this type of power-law variability in material properties to customize their responses to external influences or environmental conditions. In alignment with the design specifications and intended functionality, an exponent can be altered to effect specific changes in attributes. The FGM medium becomes homogeneous when $\:l$ equals 0. Additionally, the physical attributes in a homogeneous environment are denoted by the parameters $\:{h}_{ij}$, $\:{\rho\:}_{h}$, $\:{K}_{h}$, and $\:{K}_{h}^{*}$.

For the governing Eqs. (13), (14), (15), and (16), we can rewrite them using the relationship (17). The following are the altered forms:

18 $$\:{\tau\:}_{rr}={\left(\frac{r}{a}\right)}^{l}\left[{h}_{11}\frac{\partial\:u}{\partial\:r}+{h}_{12}\frac{2u}{r}-{\beta\:}_{1}\theta\:\right],$$

19 $$\:{\tau\:}_{\phi\:\phi\:}={\left(\frac{r}{a}\right)}^{l}\left[{h}_{12}\frac{\partial\:u}{\partial\:r}+\left({h}_{22}+{h}_{23}\right)\frac{u}{r}-{\beta\:}_{2}\theta\:\right],$$

20 $$\:\begin{array}{c}{h}_{11}\left(\frac{{\partial\:}^{2}u}{\partial\:{r}^{2}}+\frac{l+2}{r}\frac{\partial\:u}{\partial\:r}\right)+2\left[{h}_{12}\left(l+1\right)-\left({h}_{22}+{h}_{23}\right)\right]\frac{u}{{r}^{2}}\\\:-{\beta\:}_{1}\frac{\partial\:\theta\:}{\partial\:r}-\left[{\beta\:}_{1}\left(l+2\right)-2{\beta\:}_{2}\right]\frac{\theta\:}{r}={\rho\:}_{h}\frac{{\partial\:}^{2}u}{\partial\:{t}^{2}},\end{array}$$

21 $$\:\begin{array}{c}\left(1+{\tau\:}_{q}\:\frac{\partial\:}{\partial\:t}\right)\left[{\rho\:}_{h}{C}_{e}\frac{{\partial\:}^{2}\theta\:}{\partial\:{t}^{2}}+{T}_{0}\frac{{\partial\:}^{2}}{\partial\:{t}^{2}}\left({\beta\:}_{1}\frac{\partial\:u}{\partial\:r}+{\beta\:}_{2}\frac{2u}{r}\right)\right]=\\\:\left[{K}_{h}\frac{\partial\:}{\partial\:t}\left(1+{\tau\:}_{\theta\:}\:\frac{\partial\:}{\partial\:t}\right)+{K}_{h}^{*}\left(1+{\tau\:}_{\psi\:}\:\frac{\partial\:}{\partial\:t}\right)\right]\left(\frac{{\partial\:}^{2}\theta\:}{\partial\:{r}^{2}}+\frac{l+2}{r}\frac{\partial\:\theta\:}{\partial\:r}\right),\end{array}$$

where

22 $$\:{\beta\:}_{1}={\alpha\:}_{1}{h}_{11}+2{\alpha\:}_{2}{h}_{12},\:\:{\beta\:}_{2}={\alpha\:}_{1}{h}_{12}+{\alpha\:}_{2}\left({h}_{22}+{h}_{23}\right)$$

The following dimensionless variables and symbols are introduced in order to make the problem simpler:

23 $$\:\begin{array}{c}{r}^{{\prime\:}}=\frac{r}{a},\:\:\:\left\{{t}^{{\prime\:}},\:\:{{\tau\:}_{q}}^{{\prime\:}},\:\:{{\tau\:}_{\theta\:}}^{{\prime\:}},\:\:{{\tau\:}_{\psi\:}}^{{\prime\:}},\:\:{{t}_{r}}^{{\prime\:}}\right\}=\frac{v}{a}\left\{t,\:\:{\tau\:}_{q},\:\:{\tau\:}_{\theta\:},\:\:{\tau\:}_{\psi\:},\:\:{t}_{r}\right\},\:\:\\\:{u}^{{\prime\:}}=\frac{{h}_{11}}{a{T}_{0}{\beta\:}_{1}}u,\:\:\:{{\tau\:}_{ij}}^{{\prime\:}}=\frac{{\tau\:}_{ij}}{{\beta\:}_{1}{T}_{0}},\:\:\:\:{\theta\:}^{{\prime\:}}=\frac{\theta\:}{{T}_{0}},\:\:\:{{\theta\:}_{0}}^{{\prime\:}}=\frac{{\theta\:}_{0}}{{T}_{0}},\:\:\:v=\frac{{K}_{h}}{a{\rho\:}_{h}{C}_{e}}.\end{array}$$

The governing equations (18) through (21) can be rewritten in their non-dimensional forms taking into account these dimensionless variables

24 $$\:{\tau\:}_{rr}={r}^{l}\left[\frac{\partial\:u}{\partial\:r}+{\lambda\:}_{1}\frac{2u}{r}-\theta\:\right],$$

25 $$\:{\tau\:}_{\phi\:\phi\:}={r}^{l}\left[{\lambda\:}_{1}\frac{\partial\:u}{\partial\:r}+{\lambda\:}_{2}\frac{u}{r}-{\lambda\:}_{3}\theta\:\right],$$

26 $$\:\begin{array}{c}\left(1+{\tau\:}_{q}\:\frac{\partial\:}{\partial\:t}\right)\left[\frac{{\partial\:}^{2}\theta\:}{\partial\:{t}^{2}}+\:{\lambda\:}_{4}\frac{{\partial\:}^{2}}{\partial\:{t}^{2}}\left(\frac{\partial\:u}{\partial\:r}+{\lambda\:}_{3}\frac{2u}{r}\right)\right]=\\\:\left[{K}_{1}\frac{\partial\:}{\partial\:t}\left(1+{\tau\:}_{\theta\:}\:\frac{\partial\:}{\partial\:t}\right)+{K}_{2}\left(1+{\tau\:}_{\psi\:}\:\frac{\partial\:}{\partial\:t}\right)\right]\left(\frac{{\partial\:}^{2}\theta\:}{\partial\:{r}^{2}}+\frac{l+2}{r}\frac{\partial\:\theta\:}{\partial\:r}\right),\end{array}$$

27 $$\:\frac{{\partial\:}^{2}u}{\partial\:{r}^{2}}+\frac{l+2}{r}\frac{\partial\:u}{\partial\:r}-{\lambda\:}_{5}\frac{u}{{r}^{2}}-{\lambda\:}_{6}\frac{{\partial\:}^{2}u}{\partial\:{t}^{2}}=\frac{\partial\:\theta\:}{\partial\:r}+{\lambda\:}_{7}\frac{\theta\:}{r},$$

where

28 $$\:\begin{array}{c}{\lambda\:}_{1}=\frac{{h}_{12}}{{h}_{11}},\:\:\:\:{\lambda\:}_{2}=\frac{{h}_{22}+{h}_{23}}{{h}_{11}},\:\:\:{\lambda\:}_{3}=\frac{{\beta\:}_{2}}{{\beta\:}_{1}},\:\:\:{K}_{1}=\frac{{K}_{h}}{{av\rho\:}_{h}{C}_{e}},\:\:\:{K}_{2}=\frac{{K}_{h}^{*}}{{{v}^{2}\rho\:}_{h}{C}_{e}},\:\\\:\:{\lambda\:}_{4}=\frac{{{T}_{0}\beta\:}_{1}^{2}}{{h}_{11}{\rho\:}_{h}{C}_{e}},\:\:{\lambda\:}_{5}=2\left[{{\lambda\:}_{2}-\lambda\:}_{1}\left(l+1\right)\right],\:\:{\lambda\:}_{6}=\frac{{{v}^{2}\rho\:}_{h}}{{h}_{11}},\:\:{\lambda\:}_{7}=-2{\lambda\:}_{3}+l+2.\end{array}$$

### Transformed solution

Partial differential equations in the space-time domain can be resolved using the Laplace transform technique, which converts them into ordinary differential equations in the spatial domain. This modification streamlines the governing equations of the problem, hence aiding in the quest for solutions. To effectively employ the Laplace transformation approach, the subsequent prerequisites will be considered:

29 $$\:\begin{array}{c}u\left(r,t\right)=0,\:\:\frac{\partial\:u\left(r,t\right)}{\partial\:t}=0,\:\:\frac{{\partial\:}^{2}u\left(r,t\right)}{\partial\:{t}^{2}}=0,\:\:\:\:\:\:\:\text{a}\text{t}\:\:\:\:\:t=0,\\\:\theta\:\left(r,t\right)=0,\:\:\:\frac{\partial\:\theta\:\left(r,t\right)}{\partial\:t}=0,\:\:\frac{{\partial\:}^{2}\theta\:\left(r,t\right)}{\partial\:{t}^{2}}=0,\:\:\:\:\:\text{a}\text{t}\:\:\:\:\:t=0.\end{array}$$

It is possible to convert the governing equations (24) through (27) into ordinary differential equations in the Laplace domain by applying the Laplace transform

30 $$\:\stackrel{-}{{\tau\:}_{rr}}={r}^{l}\left[\frac{\text{d}\stackrel{-}{u}}{\text{d}r}+{\lambda\:}_{1}\frac{2\stackrel{-}{u}}{r}-\stackrel{-}{\theta\:}\right],$$

31 $$\:\stackrel{-}{{\tau\:}_{\phi\:\phi\:}}={r}^{l}\left[{\lambda\:}_{1}\frac{\text{d}\stackrel{-}{u}}{\text{d}r}+{\lambda\:}_{2}\frac{\stackrel{-}{u}}{r}-\frac{l+2}{2}\stackrel{-}{\theta\:}\right],$$

32 $$\:{s}^{2}\stackrel{-}{\theta\:}+\:{\lambda\:}_{4}{s}^{2}\left(\frac{\text{d}\stackrel{-}{u}}{\text{d}r}+\frac{l+2}{r}\stackrel{-}{u}\right)=\:{\lambda\:}_{8}\left(\frac{{\text{d}}^{2}\stackrel{-}{\theta\:}}{\text{d}{r}^{2}}+\frac{l+2}{r}\frac{\text{d}\stackrel{-}{\theta\:}}{\text{d}r}\right),$$

33 $$\:\frac{{\text{d}}^{2}\stackrel{-}{u}}{\text{d}{r}^{2}}+\frac{l+2}{r}\frac{\text{d}\stackrel{-}{u}}{\text{d}r}-\frac{l+2}{{r}^{2}}\frac{\stackrel{-}{u}}{r}-{\lambda\:}_{6}{s}^{2}\stackrel{-}{u}=\frac{\text{d}\stackrel{-}{\theta\:}}{\text{d}r},$$

where we suppose that $\:{\lambda\:}_{3}=\left(l+2\right)/2$, $\:{\lambda\:}_{5}=l+2$, and $\:{\lambda\:}_{8}=\frac{{K}_{1}\text{s}\left(1+{\tau\:}_{\theta\:}\:\text{s}\right)+{K}_{2}\left(1+{\tau\:}_{\psi\:}\:\text{s}\right)}{1+{\tau\:}_{q}s}$.

Next, we’ll present a new function $\:{\Psi\:}$ that is defined by the relationship:

34 $$\:\stackrel{-}{u}=\frac{d\stackrel{-}{{\Psi\:}}}{dr}$$

Equations (32) and (33), using this new function, can be rewritten as follows:

35 $$\:\left({\text{D}}_{1}{\text{D}}_{2}-{\lambda\:}_{6}{s}^{2}\right)\stackrel{-}{{\Psi\:}}=\stackrel{-}{\theta\:},$$

36 $$\:\:\frac{{\lambda\:}_{4}{s}^{2}}{{\lambda\:}_{8}}{\text{D}}_{1}{\text{D}}_{2}\stackrel{-}{{\Psi\:}}=\:\left({\text{D}}_{1}{\text{D}}_{2}-\frac{{s}^{2}}{{\lambda\:}_{8}}\right)\stackrel{-}{\theta\:},$$

where

37 $$\:{\text{D}}_{1}=\frac{\text{d}}{\text{d}r}+\frac{l+2}{r},\:\:\:{\text{D}}_{2}=\frac{\text{d}}{\text{d}r}.$$

From equations (35), and (36), we obtain the following equation:

38 $$\:\left({\text{D}}_{1}{\text{D}}_{2}-{b}_{1}^{2}\right)\left({\text{D}}_{1}{\text{D}}_{2}-{b}_{2}^{2}\right)\stackrel{-}{{\Psi\:}}=0,$$

where the following characteristic equation is satisfied by $\:{b}_{1}^{2}$ and $\:{b}_{2}^{2}$:

39 $$\:{b}^{4}-\left({\lambda\:}_{6}+\frac{1+{\lambda\:}_{4}}{{\lambda\:}_{8}}\right){s}^{2}{b}^{2}+\frac{{\lambda\:}_{6}{s}^{4}}{{\lambda\:}_{8}}=0.$$

A new dependent function $\:\stackrel{-}{{\Phi\:}}$ is introduced in order to convert Eq. (38) into a modified Bessel equation. It is defined as follows:

40 $$\:\stackrel{-}{{\Psi\:}}={r}^{-\eta\:}\stackrel{-}{{\Phi\:}},\:\:\:\:\:\:\:l=2\eta\:-1.$$

Equation (38) can be expressed in the following way after $\:\stackrel{-}{{\Psi\:}}$ is substituted:

41 $$\:\left(\frac{{\text{d}}^{2}}{\text{d}{r}^{2}}+\frac{1}{r}\frac{\text{d}}{\text{d}r}-\left(\frac{{\eta\:}^{2}}{{r}^{2}}+{b}_{1}^{2}\right)\right)\left(\frac{{\text{d}}^{2}}{\text{d}{r}^{2}}+\frac{1}{r}\frac{\text{d}}{\text{d}r}-\left(\frac{{\eta\:}^{2}}{{r}^{2}}+{b}_{2}^{2}\right)\right)\stackrel{-}{{\Phi\:}}=0.$$

The generic solution for function $\:\stackrel{-}{{\Psi\:}}$ can be written as follows:

42 $$\:\stackrel{-}{{\Psi\:}}={r}^{-\eta\:}\sum\:_{i=1}^{2}\left[{A}_{i}{K}_{\eta\:}\left({b}_{i}r\right)+{B}_{i}{I}_{\eta\:}\left({b}_{i}r\right)\right],$$

where the second- and first-kind modified Bessel functions are denoted by $\:{K}_{\eta\:}\left({b}_{i}r\right)$ and $\:{I}_{\eta\:}\left({b}_{i}r\right)$, respectively. Also integral parameters are the coefficients $\:{A}_{i}$ and $\:{B}_{i}$ (for $\:i=1$, 2).

$\:{B}_{i}=0$ is set to provide regularity and continuity within the spherical cavity. So, the answer is as follows:

43 $$\:\stackrel{-}{{\Psi\:}}={r}^{-\eta\:}\sum\:_{i=1}^{2}{A}_{i}{K}_{\eta\:}\left({b}_{i}r\right).$$

Equations (34) and (42) allow us to relate the radial displacement $\:\stackrel{-}{u}$ in the following way:

44 $$\:\stackrel{-}{u}=-{r}^{-\eta\:}\sum\:_{i=1}^{2}{A}_{i}{{b}_{i}K}_{\eta\:+1}\left({b}_{i}r\right).$$

We use Eq. (43) in place of Eq. (35) to explain the temperature $\:\stackrel{-}{\theta\:}$. The following is one way to express the temperature:

45 $$\:\stackrel{-}{\theta\:}={r}^{-\eta\:}\sum\:_{i=1}^{2}{A}_{i}\left[{b}_{i}^{2}{K}_{\eta\:+2}\left({b}_{i}r\right)-\frac{2\left(\eta\:+1\right)}{r}{b}_{i}{K}_{\eta\:+1}\left({b}_{i}r\right)-{\lambda\:}_{6}{s}^{2}{K}_{\eta\:}\left({b}_{i}r\right)\right].$$

By replacing the functions $\:\stackrel{-}{u}$ and $\:\stackrel{-}{\theta\:}$ in the governing Eqs. (30) and (31), the stress components can be obtained:

46 $$\:\stackrel{-}{{\tau\:}_{rr}}={r}^{\eta\:-1}\sum\:_{i=1}^{2}{A}_{i}\left[\frac{2\eta\:+1-2{\lambda\:}_{1}}{r}{b}_{i}{K}_{\eta\:+1}\left({b}_{i}r\right)+{\lambda\:}_{6}{s}^{2}{K}_{\eta\:}\left({b}_{i}r\right)\right],$$

47 $$\:\begin{array}{c}\stackrel{-}{{\tau\:}_{\phi\:\phi\:}}={r}^{\eta\:-1}\sum\:_{i=1}^{2}{A}_{i}\left(\left({\lambda\:}_{1}-\eta\:-\frac{1}{2}\right){b}_{i}^{2}{K}_{\eta\:+2}\left({b}_{i}r\right)+\right.\\\:\left.\frac{\left(\eta\:+1\right)\left(2\eta\:+1\right)-{\lambda\:}_{1}-{\lambda\:}_{2}}{r}{b}_{i}{K}_{\eta\:+1}\left({b}_{i}r\right)+\left(\eta\:+\frac{1}{2}\right){\lambda\:}_{6}{s}^{2}{K}_{\eta\:}\left({b}_{i}r\right)\right).\end{array}$$

Using the boundary conditions given in Eq. (8) and the Laplace transform, we obtain

48 $$\:\begin{array}{c}\stackrel{-}{{\tau\:}_{rr}}\left(r,s\right)=0\:\:\:\:\:\:\:\:\:\:\:\:\:\text{a}\text{t}\:\:\:\:r=1,\:\:\\\:\stackrel{-}{\theta\:}(r,\:s)=\frac{{\theta\:}_{0}\left(1-{e}^{-{t}_{r}s}\right)}{{t}_{r}{s}^{2}}\:\:\:\:\text{a}\text{t}\:\:\:\:r=1.\end{array}$$

The coefficients $\:{A}_{1}$ and $\:{A}_{2}$ will be found using Eqs. (45), (46), and (48) to obtain

49 $$\:\begin{array}{c}\sum\:_{i=1}^{2}{A}_{i}\left[{b}_{i}^{2}{K}_{\eta\:+2}\left({b}_{i}\right)-2\left(\eta\:+1\right){b}_{i}{K}_{\eta\:+1}\left({b}_{i}\right)-{\lambda\:}_{6}{s}^{2}{K}_{\eta\:}\left({b}_{i}\right)\right]=\frac{{\theta\:}_{0}\left(1-{e}^{-{t}_{r}s}\right)}{{t}_{r}{s}^{2}},\\\:\sum\:_{i=1}^{2}{A}_{i}\left[(2\eta\:+1-2{\lambda\:}_{1}){b}_{i}{K}_{\eta\:+1}\left({b}_{i}\right)+{\lambda\:}_{6}{s}^{2}{K}_{\eta\:}\left({b}_{i}\right)\right]=0.\end{array}$$

The linear system can be solved to find the constants $\:{A}_{1}$ and $\:{A}_{2}$.

### Special cases

Equation (21) illustrates the proposed three-phase-lag functionally graded thermal conductivity model (FGM-TPL), from which the following previous thermoelastic theories can be deduced as separate states:

- When $\:{K}_{h}^{*}=0$ and $\:{\tau\:}_{q}={\tau\:}_{\theta\:}=0$, the functionally graded conventional thermoelasticity theory (FGM-CTE) is applied. The heat equation will take the following form:

50 $$\:{\rho\:}_{h}{C}_{e}\frac{\partial\:\theta\:}{\partial\:t}+{T}_{0}\frac{\partial\:}{\partial\:t}\left({\beta\:}_{1}\frac{\partial\:u}{\partial\:r}+{\beta\:}_{2}\frac{2u}{r}\right)={K}_{h}\left(\frac{{\partial\:}^{2}\theta\:}{\partial\:{r}^{2}}+\frac{l+2}{r}\frac{\partial\:\theta\:}{\partial\:r}\right).$$

- When $\:{\tau\:}_{\theta\:}=0$ and $\:{K}_{h}^{*}=0$, the functionally graded Lord-Shulman theory (FGM-LS) is used. The formula for heat will be

51 $$\:\left(1+{\tau\:}_{q}\:\frac{\partial\:}{\partial\:t}\right)\left[{\rho\:}_{h}{C}_{e}\frac{\partial\:\theta\:}{\partial\:t}+{T}_{0}\frac{\partial\:}{\partial\:t}\left({\beta\:}_{1}\frac{\partial\:u}{\partial\:r}+{\beta\:}_{2}\frac{2u}{r}\right)\right]={K}_{h}\left(\frac{{\partial\:}^{2}\theta\:}{\partial\:{r}^{2}}+\frac{l+2}{r}\frac{\partial\:\theta\:}{\partial\:r}\right).$$

- One can derive the functionally graded Green and Naghdi third type model (FGM-GNIII) when $\:{\tau\:}_{q}={\tau\:}_{\theta\:}={\tau\:}_{\psi\:}=0$ as

52 $$\:{\rho\:}_{h}{C}_{e}\frac{{\partial\:}^{2}\theta\:}{\partial\:{t}^{2}}+{T}_{0}\frac{{\partial\:}^{2}}{\partial\:{t}^{2}}\left({\beta\:}_{1}\frac{\partial\:u}{\partial\:r}+{\beta\:}_{2}\frac{2u}{r}\right)=\left({K}_{h}^{*}+{K}_{h}\frac{\partial\:}{\partial\:t}\right)\left(\frac{{\partial\:}^{2}\theta\:}{\partial\:{r}^{2}}+\frac{l+2}{r}\frac{\partial\:\theta\:}{\partial\:r}\right).$$

- The dual-phase-lag functionally graded thermal conductivity model (FGM-DPL) is derived by putting $\:{K}_{h}^{*}=0$. The equation for heat will be

53 $$\:\left(1+{\tau\:}_{q}\:\frac{\partial\:}{\partial\:t}\right)\left[{\rho\:}_{h}{C}_{e}\frac{\partial\:\theta\:}{\partial\:t}+{T}_{0}\frac{\partial\:}{\partial\:t}\left({\beta\:}_{1}\frac{\partial\:u}{\partial\:r}+{\beta\:}_{2}\frac{2u}{r}\right)\right]={K}_{h}\left(1+{\tau\:}_{\theta\:}\:\frac{\partial\:}{\partial\:t}\right)\left(\frac{{\partial\:}^{2}\theta\:}{\partial\:{r}^{2}}+\frac{l+2}{r}\frac{\partial\:\theta\:}{\partial\:r}\right).$$

### Numerical inversion of the Laplace transform

The inverse translation of Laplace transforms can sometimes be performed analytically or by referencing established tables; however, identifying the inverse functions poses considerable challenges. A primary problem is the intricacy of inverse functions, which can be difficult to discern due to their often ambiguous nomenclature or representations. Moreover, when the Laplace transform is computed along the real and positive axes, numerous time-domain functions may provide identical Laplace transforms, resulting in ambiguity and perhaps rendering the original problem ill-posed. Moreover, the process gets increasingly complex for non-standard functions owing to the absence of a universal inversion formula. To surmount these challenges, the inverse Laplace transform is frequently computed numerically. One popular method is the Riemann-sum approximation, which uses discrete sums to evaluate the complex integral. Additional numerical techniques in the literature include Talbot’s Method, which employs contour integration to provide precise results for a range of functions; Post’s Method, which is tailored for specific classes of Laplace transforms; and numerical inversion in which the inverse transform is efficiently computed using Fast Fourier Transform (FFT) techniques^42,43^. A function $\:\stackrel{-}{g}\left(r,s\right)$ in the Laplace domain can be transformed into a physical field function $\:g\left(r,t\right)$ using the following relation:

54 $$\:g\left(r,t\right)=\frac{{e}^{\varsigma\:t}}{2t}Re\left\{\stackrel{-}{g}\left(r,\varsigma\:\right)+2{\sum\:}_{n=1}^{k}\stackrel{-}{g}\left(r,\varsigma\:+\frac{in\pi\:}{t}\right){\left(-1\right)}^{n}\right\},$$

where the imaginary unit is represented by $\:i=\sqrt{-1}$ and the real component is indicated by $\:Re$.

The convergence of the series is contingent upon the behavior of the function $\:\stackrel{-}{g}\left(r,\varsigma\:\right)$ and its values as $\:n$ escalates. If $\:\stackrel{-}{g}\left(r,\varsigma\:\right)$ is continuous and well-defined, the series is expected to converge as $\:k$ rises. The terms $\:\stackrel{-}{g}\left(r,\varsigma\:+\frac{in\pi\:}{t}\right){\left(-1\right)}^{n}$ must diminish rapidly enough for the summation to converge. Should the terms not diminish swiftly, additional terms will be requisite for attaining a precise approximation. For numerical analyses, the value of ς is chosen such that $\:\varsigma\:t\cong\:4.7$ to ensure rapid convergence^42^. To determine the requisite number of phrases, we can evaluate the error. The error in the Riemann sum can frequently be approximated by assessing the amount of the final term incorporated in the sum. If the terms diminish in magnitude such that the absolute value of $\:\stackrel{-}{g}\left(r,\varsigma\:+\frac{in\pi\:}{t}\right)$ approaches 0, a threshold can be established for the error to ascertain the requisite number of terms k. For the majority of issues, a moderate value of $\:k$ (e.g., $\:k\cong\:10$) is typically adequate^43^. In a number of applications, this particular value has been demonstrated to have positive outcomes. This computational method is renowned for its efficacy and efficiency, especially when used to functions with smooth characteristics. Frequently, the approach yields trustworthy outcomes in numerical calculations.

## Results and discussion

In this section, the thermo-elastic behavior of titanium alloy is the main focus of the discussion and numerical results obtained from the theoretical framework. Titanium alloys are metallic materials composed primarily of titanium, combined with other elements to enhance their properties. Known for their exceptional strength-to-weight ratio, they are lightweight yet incredibly strong, making them ideal for demanding applications. Additionally, titanium alloys exhibit excellent corrosion resistance, enabling them to withstand harsh environments. Their biocompatibility also makes them suitable for medical implants. These properties contribute to their widespread use in industries such as aerospace, automotive, medical, and marine, where performance and durability are critical. By investigating titanium alloy’s thermo-elastic behavior, this study seeks to clarify its possible advantages and uses in a range of engineering domains.

The supplied physical data for titanium alloy is used in numerical computations to produce the numerical results. The following material and physical constants were utilized in the numerical calculations for titanium alloy^44^:

$$\:{h}_{11}=17.44\times\:{10}^{9}\:\frac{\text{N}}{{\text{m}}^{2}},\:\:{h}_{12}=6.17\times\:{10}^{9}\:\frac{\text{N}}{{\text{m}}^{2}},\:\:{h}_{23}=4.96\times\:{10}^{9}\:\frac{\text{N}}{{\text{m}}^{2}},\:{\theta\:}_{0}=0.3,$$

$$\:{K}_{h}^{*}=200\:\frac{\text{W}}{\text{s}\:\text{m}\:\text{K}},\:\:{\alpha\:}_{1}=15\times\:{10}^{-6}\:\frac{1}{\text{K}},\:\:{\alpha\:}_{2}=23\times\:{10}^{-6}\:\frac{\:1}{\text{K}},\:{K}_{h}=0.918\:\frac{\text{W}}{\text{m}\:\text{K}},{C}_{e}=262\:\frac{\text{J}}{\text{k}\text{g}\:\text{K}},$$

$$\:{T}_{0}=200.15\:\text{K},\:{\tau\:}_{q}=0.3,\:{\tau\:}_{\theta\:}=0.2,\:\:{\tau\:}_{\psi\:}=0.1,\:\:a=0.01\:\text{m},\:\:l=2,\:\:t=0.07,\:\:{\rho\:}_{h}=7960\:\frac{\text{k}\text{g}}{{\text{m}}^{3}}.$$

The displacement $\:u$, temperature variation $\:\theta\:$, radial stress $\:{\tau\:}_{rr}$, and hoop stress $\:{\tau\:}_{\vartheta\:\vartheta\:}$ along the radial axis $\:r$ are among the system variables that will be numerically calculated. A number of contemporary theories of thermoelasticity will be compared to the current model in order to verify its accuracy. In particular, the effects of ramping time will be examined in a comparison study. Mathematica will be used to generate the computations, numerical results, and graphical representations. This methodology will provide a thorough examination of the linked behavior within the system and successfully validate the suggested model.

The outcomes confirm that the suggested model is accurate and efficient in capturing the coupled thermo-elastic behavior of a hollow sphere in a functionally graded infinite medium. The research demonstrates how the system variables are significantly impacted by ramping time, relaxation time, and thermal conductivity rate. These results provide crucial information for the design and optimization of functionally graded structures in a range of applications, such as electronics, energy systems, automotive, aerospace, and thermal management systems. Through comprehension of these connections, engineers might improve the functionality and dependability of materials utilized in these vital sectors.

This first part will present a comparison analysis between the proposed model and previous thermoelastic models, which are dissimilar from the current model (see Sect. 6). The ramping time $\:{t}_{r}=0.08$ is employed in numerical calculations. The results indicate a distinct differentiation among the outputs of the FGM-CTE, FGM-LS, FGM-GNIII, FGM-DPL, and FGM-TPL models. The relaxation times $\:{\tau\:}_{q}$, $\:{\tau\:}_{\theta\:}$, and $\:{\tau\:}_{\psi\:}$ considerably affect the various field distributions.

**Fig. 2:**
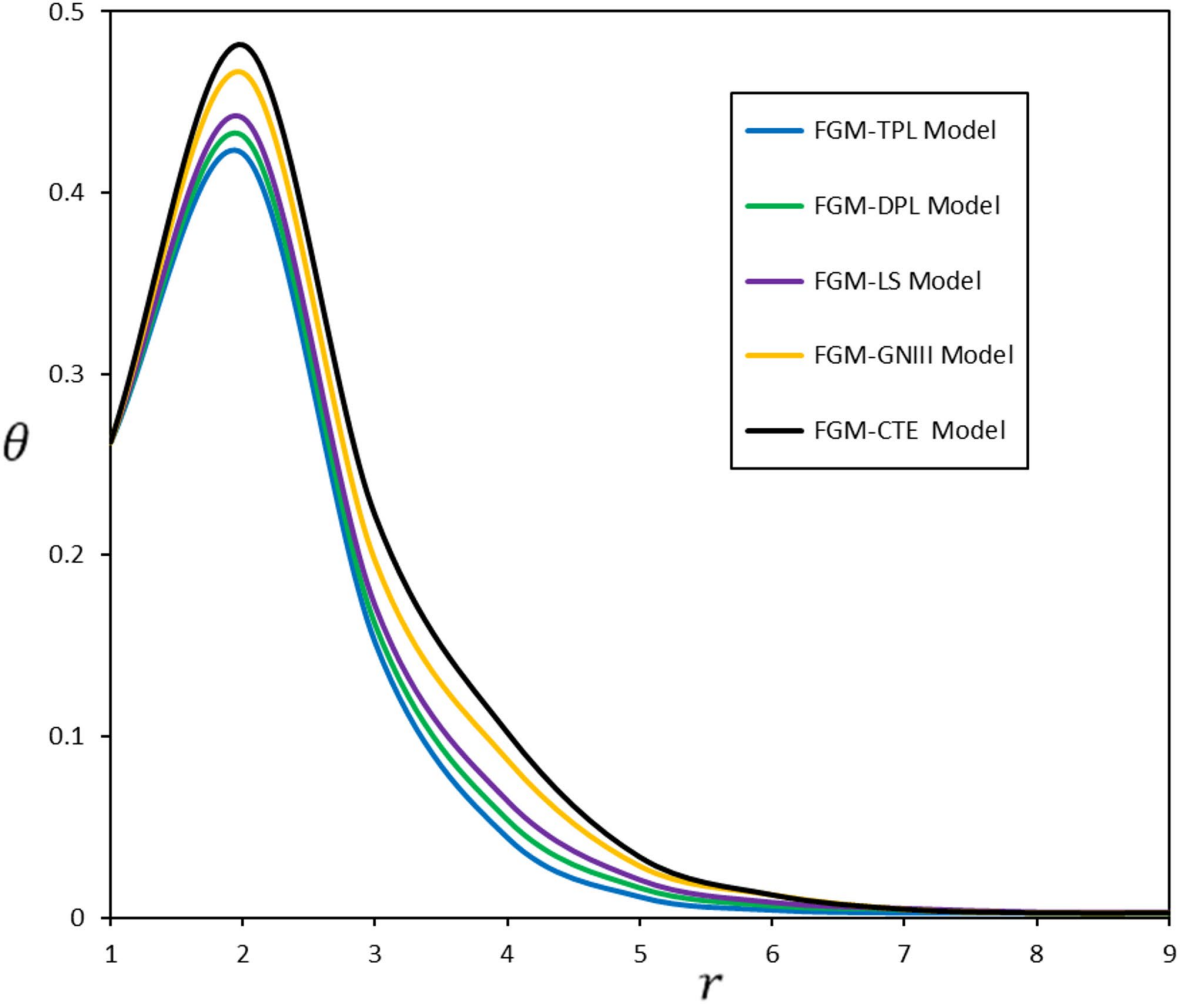
Diverse thermoelastic models and their impact on temperature change $\:\theta\:$

Figure 2 illustrates the fluctuation of thermodynamic temperature $\:\theta\:$ in relation to the radius r across various thermoelasticity models. The graph demonstrates that the FGM-GNIII and FGM-CTE models, which do not incorporate relaxation times ($\:{\tau\:}_{q}$, $\:{\tau\:}_{\theta\:}$, and $\:{\tau\:}_{\psi\:})$, have elevated temperature values in contrast to the FGM-LS, FGM-DPL, and FGM-TPL models. An essential factor to examine in this context is the convergence of the outcomes from the FGM-GNIII and FGM-CTE models. This convergence prompted the creation of the FGM-GNIII model and the implementation of the present model.

Heat waves propagate at a slower rate in the FGM-DPL and FGM-TPL models than in the FGM-LS model. The disparity can be ascribed to the time delay $\:{\tau\:}_{\theta\:}$ included in both revised models. The incorporation of this characteristic induces a temporal delay in the material’s response to temperature fluctuations. This observation further validates the precision of the results generated by the proposed model, which provides a more authentic depiction of thermal wave propagation across materials with diverse properties.

**Fig. 3:**
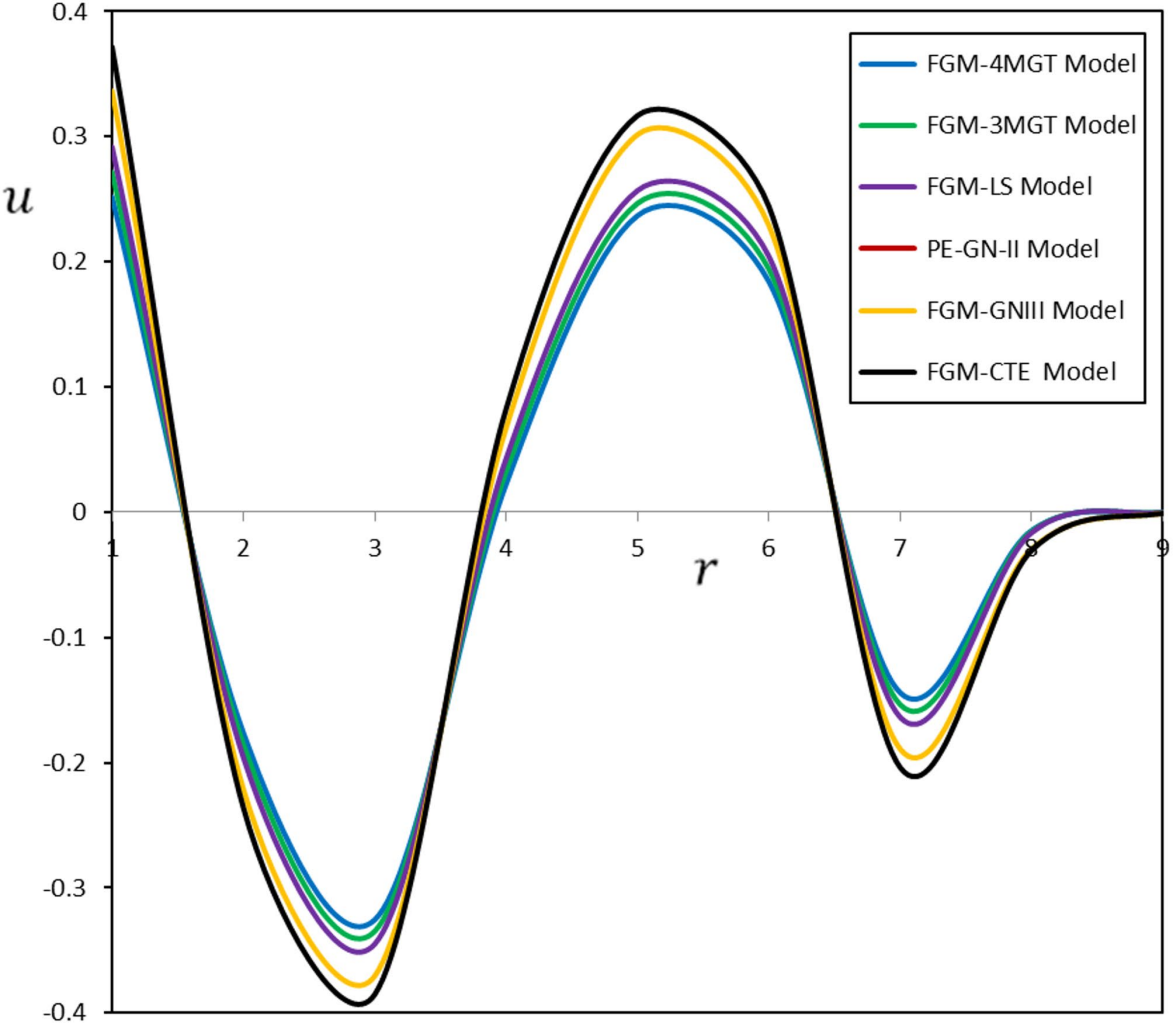
Diverse thermoelastic models and their impact on displacement $\:u$

**Fig. 4:**
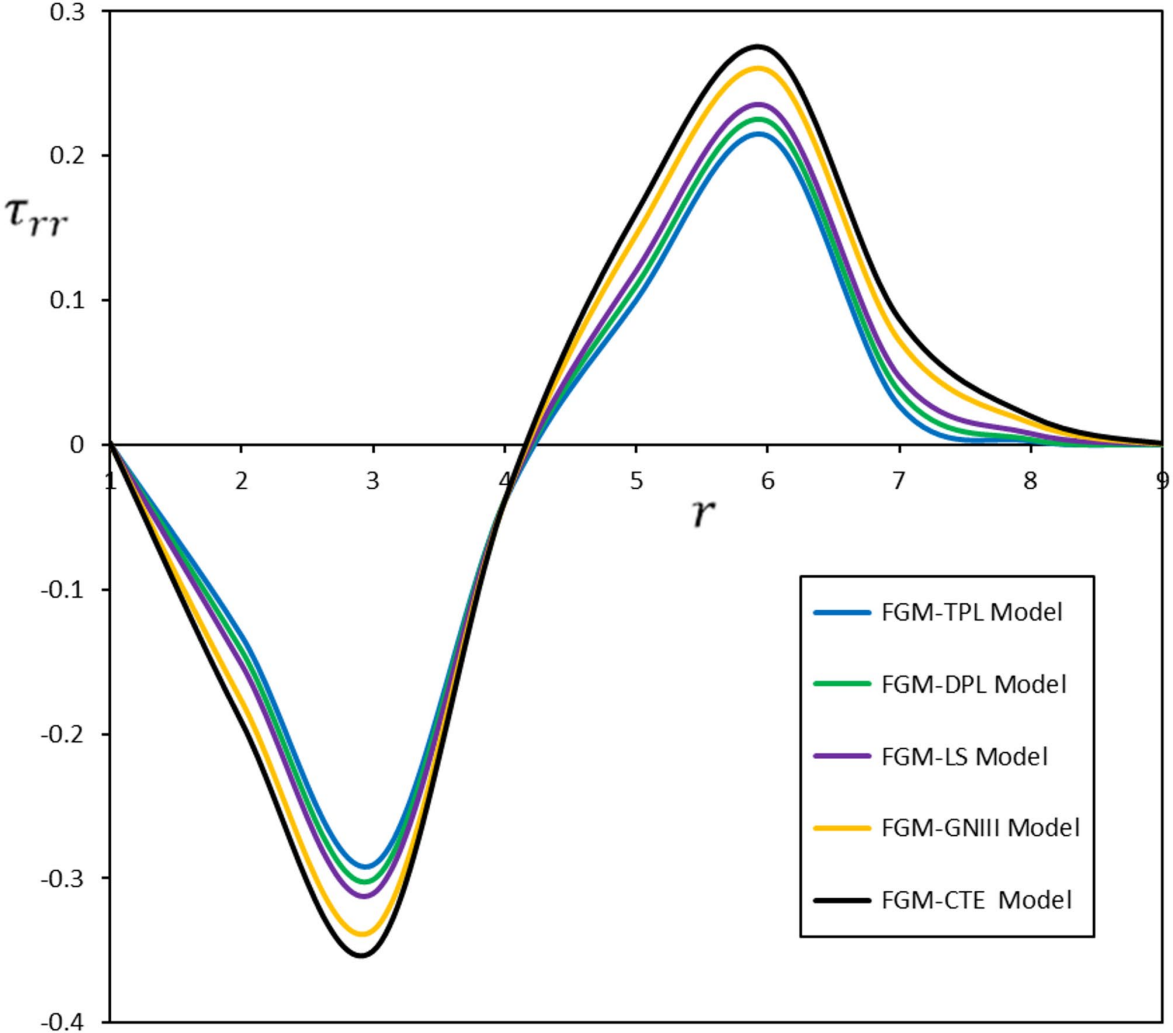
Diverse thermoelastic models and their impact on radial stress $\:{\tau\:}_{rr}$

**Fig. 5:**
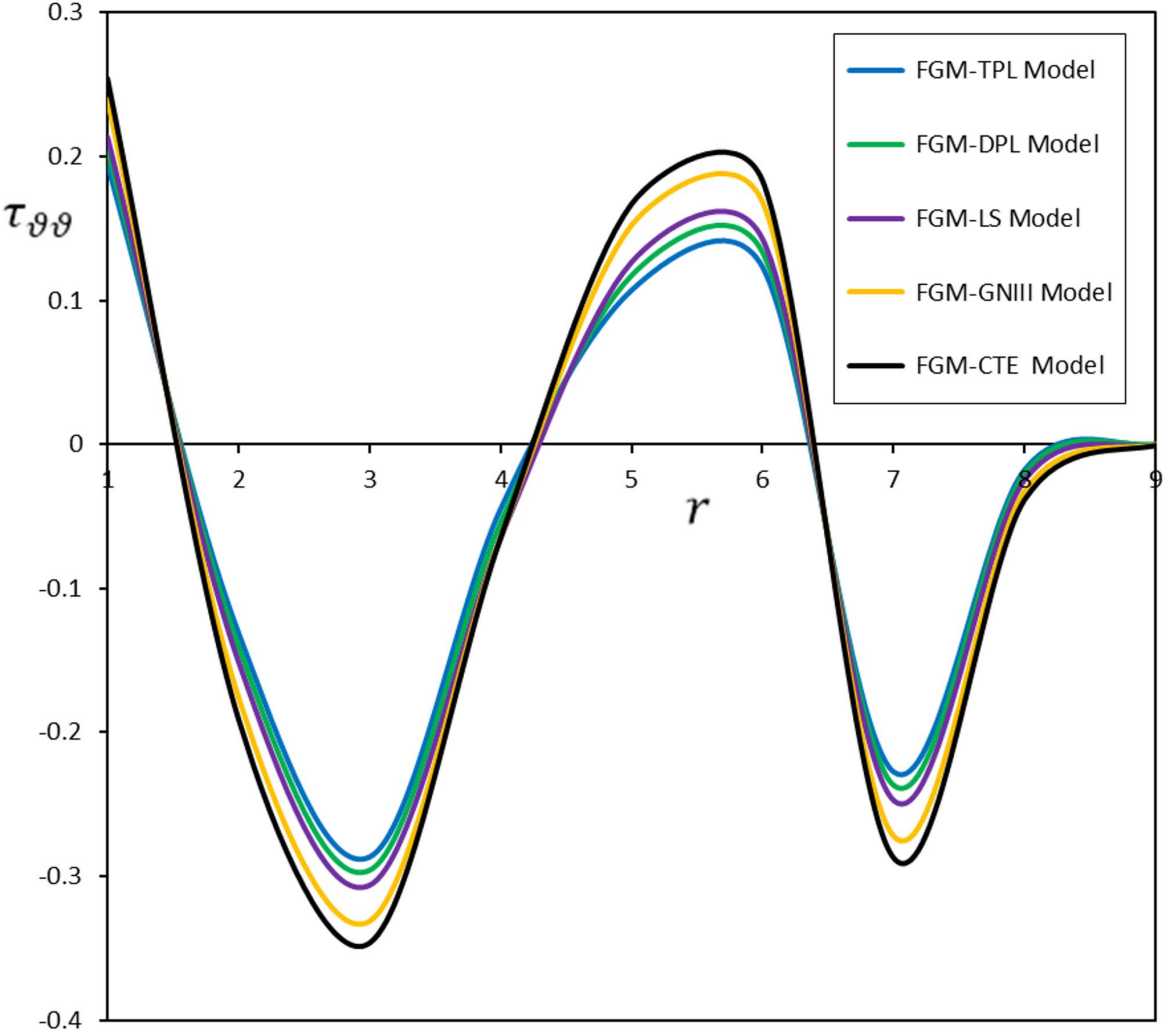
Diverse thermoelastic models and their impact on hoop stress $\:{\tau\:}_{\vartheta\:\vartheta\:}$

The fluctuations in displacement $\:u$, radial stress $\:{\tau\:}_{rr}$, and hoop stress $\:{\tau\:}_{\vartheta\:\vartheta\:}$ as functions of radial distance ($\:r$) are depicted in Figs. 3 and 4, and 5. The delay induced by the time delay $\:{\tau\:}_{\theta\:}$ substantially influences the temporal progression of deformation and stress in the material. The displacement ($\:u$) measured in the FGM-DPL and FGM-TPL models demonstrates a lagged temperature response relative to conventional models. Furthermore, the thermal stresses ($\:{\tau\:}_{rr}$, $\:{\tau\:}_{\vartheta\:\vartheta\:}$) exhibit shallower gradients when examined using the FGM-DPL and FGM-TPL models. The results correspond with observations documented in the literature^45,46^offering further confirmation and comparative analysis of the behavior of diverse thermoelastic models.

Upon comparison of the FGM-DPL and FGM-TPL models, it is evident that the FGM-TPL model enhances accuracy by integrating the time delay $\:{\tau\:}_{\psi\:}$, which more precisely accounts for heat diffusion over time, thereby yielding a more realistic depiction of stresses and displacements.

The FGM-TPL model offers significant advantages because of its extensive framework for investigating thermoelastic behavior. They effectively integrate thermal relaxation effects and account for higher-order spatial and temporal gradients, enabling precise predictions of distributed deformation and stress fields. Conversely, classical models such as the FGM-CTE and FGM-GNIII are less successful for materials exhibiting thermal relaxation or higher-order effects, frequently resulting in overestimations of stress and deformation while disregarding time-dependent thermal reactions.

The incorporation of a thermal relaxation delay improves the thermoelastic response, resulting in a time-dependent behavior affected by both present conditions and previous temperature variations. This dynamic behavior is defined by fluctuations in displacement and thermal stresses, influenced by the material’s characteristics, the nature of the applied thermal stress, and the duration of the relaxation period. These parameters must be meticulously evaluated when assessing materials subjected to heat loading to precisely forecast their long-term performance.

Thermal stimulation alters thermophysical variables, and the finite velocity of thermal impulses results in wave-like heat propagation through the material, adhering to the temperature profile. This aspect highlights the significance of acknowledging constrained heat transport velocities in sophisticated thermoelastic models, which more accurately depict actual thermal responses in materials. The findings offer significant insights into the interaction of thermal relaxation, material characteristics, and dynamic thermoelastic behavior, enhancing comprehension of heat-induced phenomena in engineering contexts.

The ramifications of this study are substantial for practical applications. The FGM-TPL model is very advantageous for studying materials and structures where thermal relaxation is critical, notably in microscale and nanoscale systems. Moreover, they are crucial for accurately capturing stress and deformation distributions in scenarios involving thin-walled structures and heat-source heating systems, hence improving the design and performance of these sophisticated materials.

The findings of this investigation align with previous research^47–49^confirming the precision and dependability of the utilized numerical method. All offered pictures demonstrate the phenomenon of finite thermal wave velocity, a characteristic of sophisticated thermoelastic models. The data indicate that employing a temperature-variable method at the free-stress border results in notable variations in the thermoelastic response adjacent to the boundary, which decrease with greater distance from it. This underscores the confined impact of boundary conditions and the progressive attenuation of changes further within the material.

This second part will discuss the significance of ramping time ( $\:{t}_{r}$) in functionally graded thermoelastic systems. Comprehending the impact of ramping time ($\:{t}_{r}$) provides enhanced insights into the mechanics of heat transfer and thermomechanical interaction in functionally graded thermoelastic materials. This knowledge clarifies the material’s dynamic behavior under thermal stress and improves the predicted accuracy of the models used for these investigations. By controlling ramping time, engineers and researchers can enhance the performance and dependability of functionally graded materials across many applications.

**Fig. 6:**
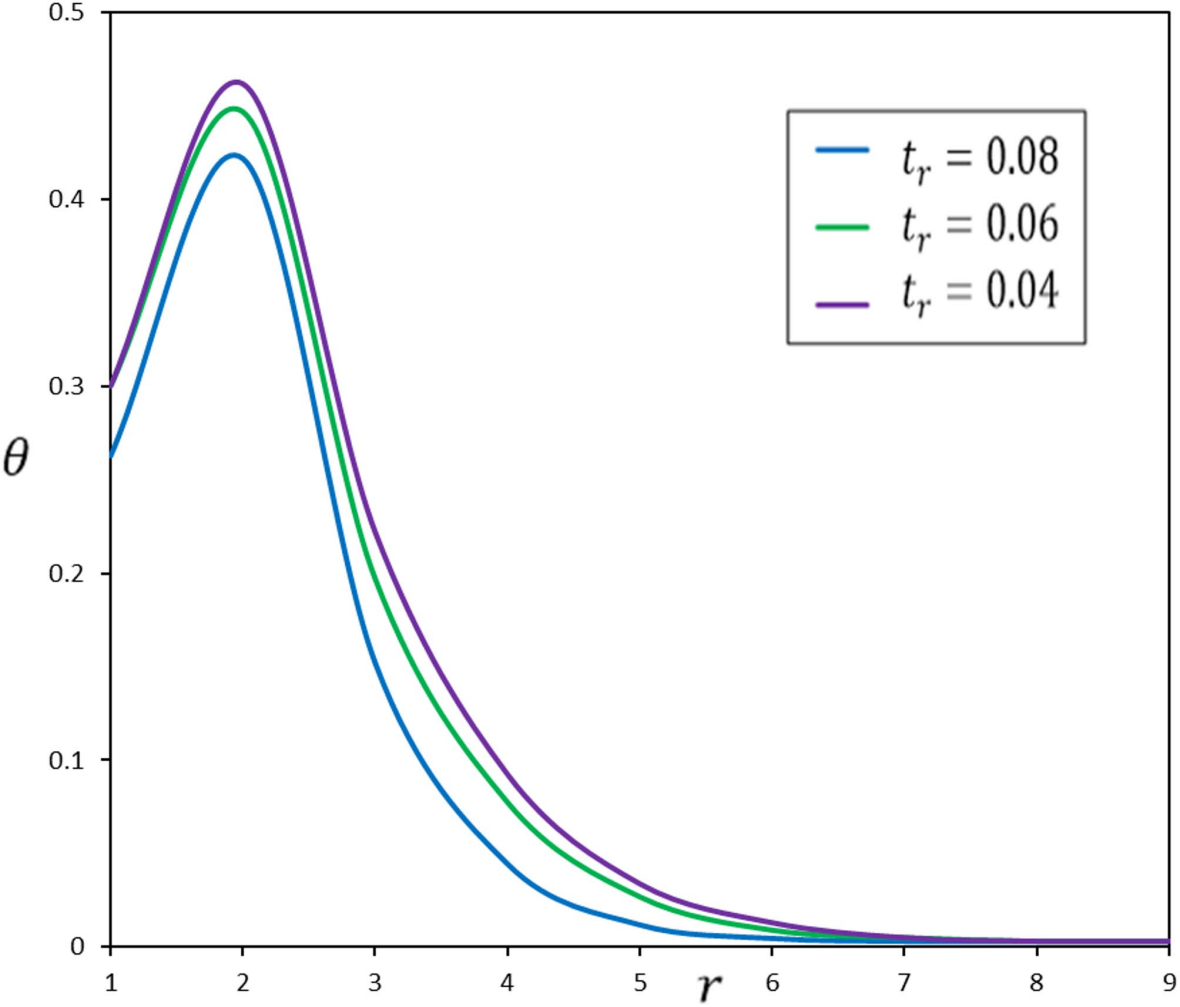
The effect of the pulse duration of heat flow, $\:{t}_{r}$, on the temperature change, $\:\theta\:$.

**Fig. 7:**
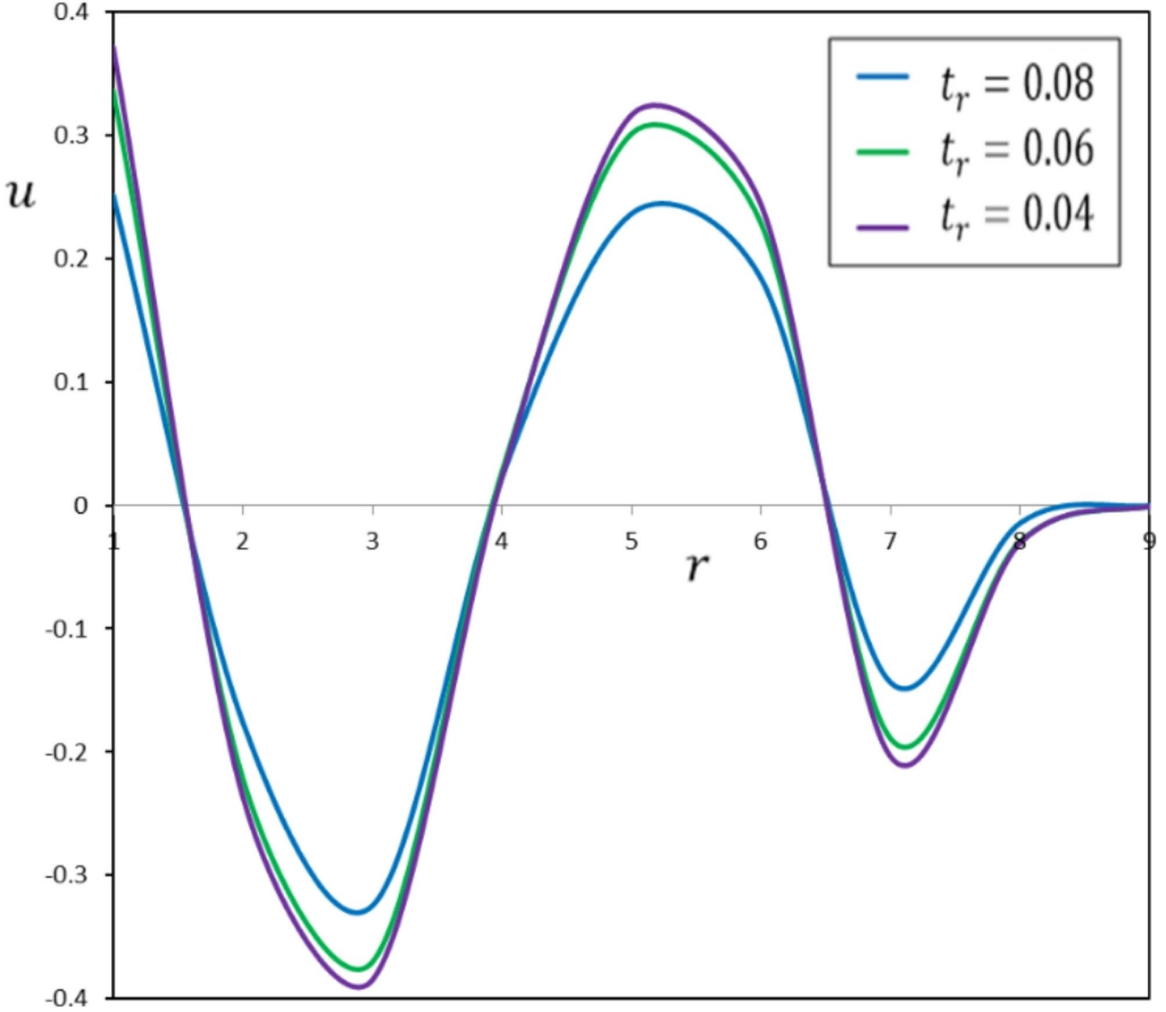
The effect of the pulse duration of heat flow, $\:{t}_{r}$, on the displacement, $\:u$.

**Fig. 8:**
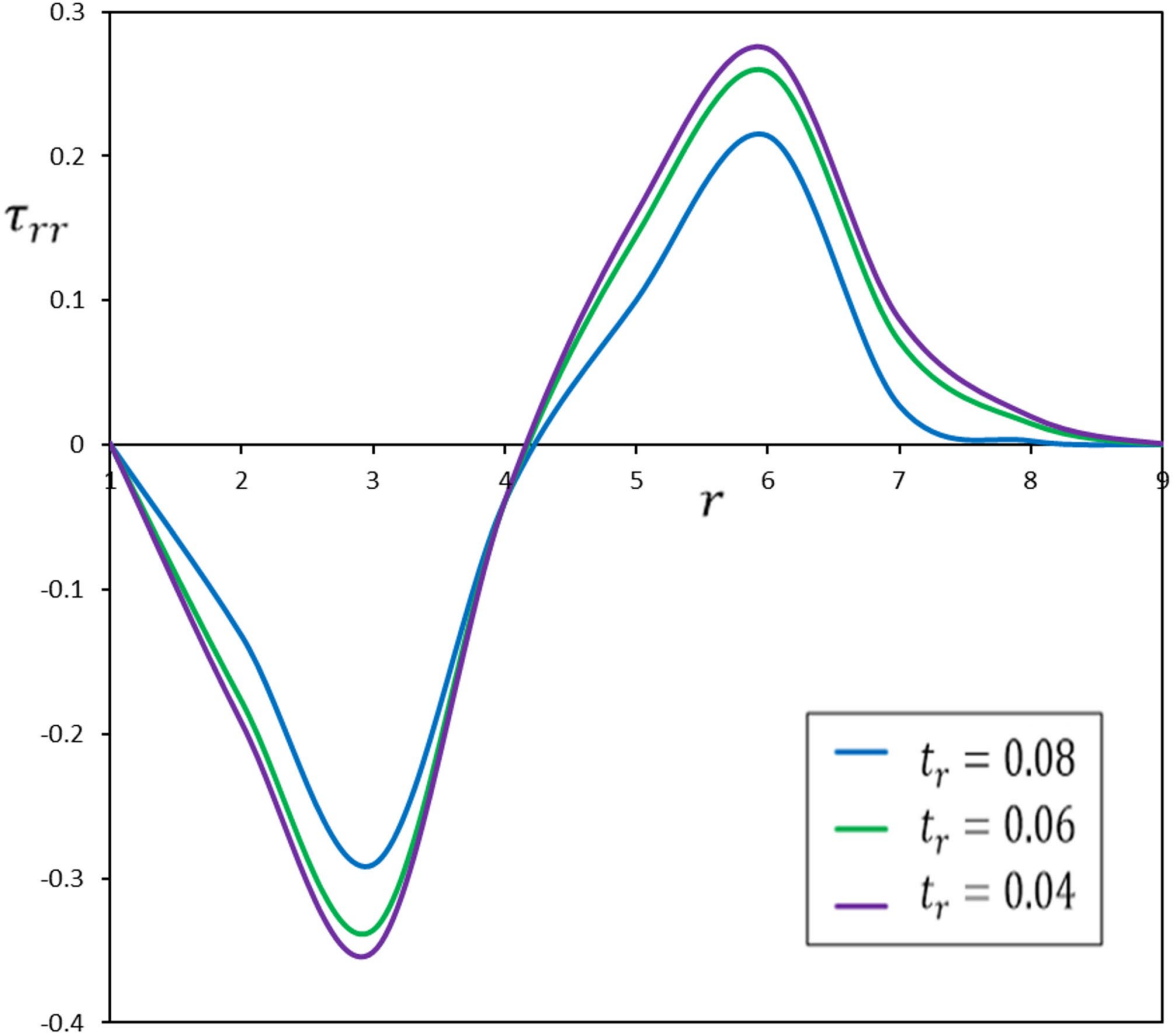
The effect of the pulse duration of heat flow, $\:{t}_{r}$, on the radial stress, $\:{\tau\:}_{rr}$.

**Fig. 9:**
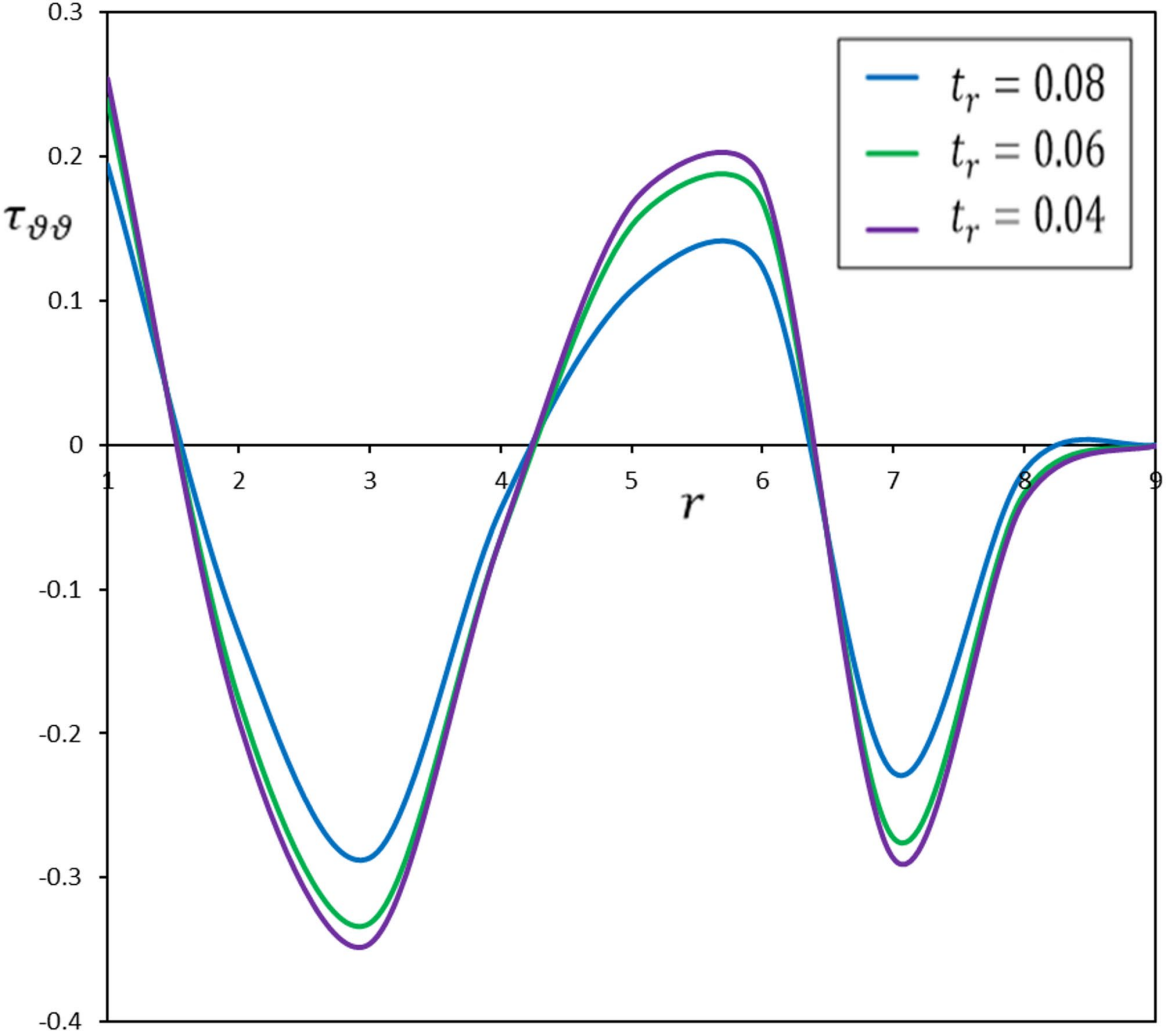
The effect of the pulse duration of heat flow, $\:{t}_{r}$, on the hoop stress, $\:{\tau\:}_{\vartheta\:\vartheta\:}$.

To examine the impact of $\:{t}_{r}$, numerical simulations were conducted for three distinct ramping times: $\:{t}_{l}$ = 0.04, 0.06, and 0.08. Figures 6, 7, 8 and 9 illustrate the impact of $\:{t}_{r}$ on the thermomechanical responses of the functionally graded thermoelastic material. This discussion offers a comprehensive analysis of the results.

The ramping time ($\:{t}_{r}$) substantially affects the thermal and mechanical reactions of the material. A reduced $\:{t}_{r}$ results in swift and localized thermal and mechanical responses, leading to increased gradients. Conversely, an extended $\:{t}_{r}$ yields more uniform and expansive distributions of temperature, displacement, and stress, resulting in diminished gradients. This signifies that the material undergoes a more progressive response across an extensive area, diminishing the probability of localized stress concentrations that may result in failure.

The kinetics of heat transmission and the behaviors of thermomechanical coupling significantly fluctuate with varying ramping times ($\:{t}_{r}$). This variance underscores the reliance of material reactions on ramping time, indicating that shorter ramping times elicit more rapid changes, whilst longer ramping times promote a more stable and uniform thermal response. Comprehending these distinctions is essential for enhancing the design and utilization of functionally graded materials, as they directly influence performance in practical applications. By customizing the ramping time for particular applications, engineers can improve the efficiency and reliability of systems dependent on accurate thermal and mechanical interactions.

A reduced $\:{t}_{r}$ correlates with an increased rate of heat deposition, resulting in swift thermal responses. The focused heating produces sharper thermal gradients, increasing the probability of temperature overshoots and transient fluctuations. Conversely, an extended $\:{t}_{r}$ diminishes the rate of heat deposition, facilitating a more uniform temperature distribution across the medium. This mitigates peak temperatures but extends the thermal impacts within the material.

The importance of these findings is considerable for multiple applications. A shorter $\:{t}_{r}$ is appropriate for situations necessitating targeted heating with pronounced thermal effects, such as in micromachining by ramp-type heating or thermal shock investigations. Conversely, an extended $\:{t}_{r}$ is optimal for operations requiring consistent heating across extensive areas, such as thermal annealing or heat treatment systems. The validation of these results against existing literature corroborates the precision of the proposed model in representing temperature dynamics across varying heating rates.

Rapid temperature variations linked to a reduced $\:{t}_{r}$ enhance thermal expansion rates, leading to significant displacements and stresses. Conversely, a gradual increase in temperature, characterized by an extended $\:{t}_{r}$, diminishes the rate of thermal expansion, resulting in minimal displacements and stresses.

These ideas hold importance for practical applications. A reduced $\:{t}_{r}$ generates significant mechanical responses appropriate for situations necessitating fast deformation $\:u$, such as thermal shock testing or ramp-type heating-assisted shaping. In contrast, an extended $\:{t}_{r}$ reduces transient displacement effects, rendering it more appropriate for preserving structural stability in systems subjected to extended heating, such as thermal energy systems.

The ramifications of these findings are significant for numerous applications. The elevated radial stresses produced by a short $\:{t}_{r}$ might result in cracks, fractures, or material failure, which is especially crucial in ramp-type heating-based manufacturing or quick thermal treatments. In contrast, extended $\:{t}_{r}$ values are preferable for applications sensitive to structural integrity, reducing stress-induced damage.

Regulating $\:{t}_{r}$ is crucial for mitigating stress-induced damage in functionally graded devices and materials exposed to ramp-type heating, particularly in applications requiring mechanical resilience. In general, reduced ramping times generate more pronounced thermal gradients, resulting in concentrated and strong thermal and mechanical reactions. These conditions yield elevated peak values for temperature, displacement, and stresses. These profiles are appropriate for applications necessitating brief, severe thermal effects; nonetheless, they may elevate the risk of material failure due to the significant stresses involved.

Conversely, extended ramping times yield smoother and more uniform distributions across all examined variables. Although they produce diminished peak values, they also generate prolonged thermal impacts. This renders them suitable for applications necessitating structural stability and slow thermal responses, guaranteeing that materials can endure extended exposure to fluctuating heat conditions without affecting their integrity.

The results are consistent with other research recorded in the literature^45,46,50,51^. These experiments further validate the suggested model and enhance the comprehension of the interplay between ramping time, thermal dynamics, and the consequent mechanical reactions in functionally graded materials. The alignment of these results with prior studies underscores the model’s robustness in precisely representing the intricate interactions that transpire during rapid heating processes.

These discoveries are crucial for comprehending and forecasting the dynamic behavior of functionally graded materials subjected to thermal stress, facilitating the design and optimization of systems in applications like ramp-type heating-based manufacturing. By customizing $\:{t}_{r}$, engineers can achieve a balance between desirable thermal effects and the reduction of stress-induced damage, hence improving the performance and reliability of functionally graded systems.

## Conclusions

This study sought to advance thermoelasticity by introducing an innovative three-phase-lag (TPL) thermoelasticity model. The three-phase-lag (TPL) equation is integrated with the Lord–Shulman, the dual-phase-lag (DPL), and Green–Naghdi type III (GN-III) thermoelastic theories in this new model. Its capacity to deconstruct into several proven theories underscores its flexibility and adaptability, indicating substantial progress in our comprehension of thermoelastic behaviour. The model was validated by analysing the thermo-elastic response of a heterogeneous transversely isotropic functionally graded material including a spherical hole, with thermophysical fields depicted visually. The research examined the impact of ramping time on thermophysical field behaviour and offers a comprehensive comparison of classical and non-classical thermoelasticity models.

The subsequent principal conclusions were derived:

This comparative study provides significant insights into the impact of changes in functionally graded material properties on thermo-mechanical responses, hence aiding in the design and optimization of heterogeneous functionally graded materials. The three-phase-lag functionally graded (FGM-TPL) model notably includes the relaxation time in heat flux, the relaxation time in temperature gradient, and the relaxation time in thermal displacement gradient, acknowledging the finite duration required for material response to temperature variations, thus introducing a temporal aspect to the heat transfer process.

The FGM-TPL model’s adaptability enables the modification of thermal parameters to conform to diverse existing thermoelasticity theories. This adaptability renders it appropriate for diverse settings, allowing it to function efficiently with both classical and non-classical models as required.

This research offers a unique contribution to the literature, as the generalised FGM-TPL thermoelastic model has not been extensively investigated in previous publications. It offers a novel viewpoint on the interplay between thermal and mechanical phenomena, representing a substantial progression in the discipline.

The overall reaction of the functionally graded material is profoundly affected by temperature variations and the ramping time. These interactions significantly influence the mechanical and thermal properties of the functionally graded material, underscoring the significance of coupling effects in functionally graded systems.

Rapid thermal responses, in physical terms, arise from the absence of relaxation times or the reduction in ramping duration, correlated with an increased rate of heat deposition. Temperature overshoots and transient fluctuations are more probable due to the concentrated heating’s establishment of steeper thermal gradients. The existence of relaxation times or the increase in ramping time, conversely, diminishes the rate of heat deposition and allows for a more uniform temperature distribution across the medium. This extends the thermal impacts of the material while reducing peak temperatures. This aligns with temperature diagrams.

Physically, significant displacements and elevated stresses result from thermal expansion rates that are exacerbated by rapid temperature fluctuations, which are associated with the absence of relaxation periods or reduced ramping durations. Conversely, a gradual temperature rise, as seen by the existence of relaxation times or the increase in ramping duration, diminishes the rate of thermal expansion, leading to reduced stresses and minimal displacements. This aligns with the displacement and stress diagrams.

The FGM-TPL model demonstrates the capabilities of functionally graded materials, which can be tailored for diverse technical and industrial applications. These applications encompass aviation components, biomedical engineering, automobile technology, and energy systems.

This research presents a generalised three-phase-lag (FGM-TPL) thermoelastic model. The model integrates temporal effects in heat conduction, providing a more precise depiction of interrelated thermal and mechanical phenomena. The model’s versatility enables it to integrate current theories while offering a new framework for analysing thermo-elastic reactions in functionally graded materials.

The results highlight the significant impact of ramping time on thermal and mechanical responses, providing essential insights for enhancing material performance in applications like ramp-type heating and the creation of sophisticated functionally graded devices. This study facilitates further investigation into the linked thermo-mechanical behaviours of heterogeneous functionally graded materials and intricate engineering systems.

## Data Availability

All available data are present in the manuscript. All data and models generated or used during the study appear in the submitted article.
